# Exogenous Calcium Enhances the Photosystem II Photochemistry Response in Salt Stressed Tall Fescue

**DOI:** 10.3389/fpls.2017.02032

**Published:** 2017-11-30

**Authors:** Guangyang Wang, Aoyue Bi, Erick Amombo, Huiying Li, Liang Zhang, Cheng Cheng, Tao Hu, Jinmin Fu

**Affiliations:** ^1^Key Laboratory of Plant Germplasm Enhancement and Specialty Agriculture, Wuhan Botanical Garden, Chinese Academy of Sciences, Wuhan, China; ^2^University of Chinese Academy of Sciences, Beijing, China; ^3^School of Resources and Environmental Engineering, Ludong University, Yantai, China

**Keywords:** exogenous calcium, PSII photochemistry, carbon and nitrogen assimilation, salt stress, tall fescue

## Abstract

Calcium enhances turfgrass response to salt stress. However, little is known about PSII photochemical changes when exogenous calcium was applied in salinity-stressed turfgrass. Here, we probe into the rearrangements of PSII electron transport and endogenous ion accumulation in tall fescue (*Festuca arundinacea* Schreber) treated with exogenous calcium under salt stress. Three-month-old seedlings of genotype “TF133” were subjected to the control (CK), salinity (S), salinity + calcium nitrate (SC), and salinity + ethylene glycol tetraacetic acid (SE). Calcium nitrate and ethylene glycol tetraacetic acid was used as exogenous calcium donor and calcium chelating agent respectively. At the end of a 5-day duration treatment, samples in SC regime had better photochemistry performance on several parameters than salinity only. Such as the Area (equal to the plastoquinone pool size), N (number of ${\text{Q}}_{\text{A}}^{-}$ redox turnovers until F_m_ is reached), ψE_0_, or δRo (Efficiencdy/probability with which a PSII trapped electron is transferred from Q_A_ to Q_B_ or PSI acceptors), ABS/RC (Absorbed photon flux per RC). All the above suggested that calcium enhanced the electron transfer of PSII (especially beyond ${\text{Q}}_{\text{A}}^{-}$) and prevented reaction centers from inactivation in salt-stressed tall fescue. Furthermore, both grass shoot and root tissues generally accumulated more C, N, Ca^2+^, and K^+^ in the SC regime than S regime. Interrelated analysis indicated that ψE_0_, δRo, ABS/RC, C, and N content in shoots was highly correlated to each other and significantly positively related to Ca^2+^ and K^+^ content in roots. Besides, high salt increased *ATP6E* and *CAMK2* transcription level in shoot at 1 and 5 day, respectively while exogenous calcium relieved it. In root, *CAMK2* level was reduced by Salinity at 5 day and exogenous calcium recovered it. These observations involved in electron transport capacity and ion accumulation assist in understanding better the protective role of exogenous calcium in tall fescue under salt stress.

## Introduction

Salinity is a major abiotic stress factor threating plant growth and crop yield (Shabala and Cuin, 2007; Türkan and Demiral, 2009). A considerable amount of fundamental processes in plant life, for instance the photosynthesis, were vulnerable with increasing salinity (Sayed, 2003; Murata et al., 2007; Chaves et al., 2009). Moreover, photosystem II (PSII) is more sensitive than photosystem I (PS I) in response to salinity (Apostolova et al., 2006). Chlorophyll *a* fluorescence transient is known as an informative tool reflecting the induced primary reaction alternations of PSII under salinity (Fricke and Peters, 2002; Sayed, 2003; Stirbet et al., 2014). In general, chlorophyll *a* fluorescence intensity shows a multiphase rise starting with at minimal level F_O_ (the O step), and terminating with the maximal level F_M_ (the P step). These two reaction points are separated by two intermediary levels denoted as F_J_ (the J step) and F_I_ (the I step) when illumination initiates on dark-adapted leaves. To investigate PSII behaviors in O-J-I-P transient, JIP test was developed to quantify the derived photochemical parameters (Strasser, 1987, 1997; Dabrowski et al., 2016). However, the PSII photochemistry response to salinity stress is still under debate. Inhibition of PSII activity was observed in maize (*Zea mays* L.; Hichem et al., 2009), Brassica species (Jamil et al., 2014), while no effect on PSII is reported in Suaeda (*Suaeda salsa* L.; Lu et al., 2003) and Rumex (*Rumex patientia* × *R. tianschaious*) (Chen et al., 2004).

Previous studies suggested that there might be potential competition between C and N assimilation on basis of sharing reducing power and carbon skeleton provided firsthand by photosynthetic electron transport and CO_2_ assimilation (Huppe and Turpin, 1994; Champigny, 1995; Sugiyama and Sakakibara, 2002; Sinclair, 2004). Nevertheless, the competitive correlativity between the two assimilations in tall fescue under salt stress has not been clearly documented by previous works.

Salinity stress leads to homeostasis imbalance in reactive oxygen species (ROS) and ion distribution (Zhu, 2001, 2002). Cytotoxic ROS are prone to trigger lipid peroxidation reactions, disrupt cellular membrane integrity and increase electrolyte leakage. An imbalance between efflux and influx induces K^+^ deficit which negatively impact stoma conductance (Fischer and Hsiao, 1968; Çavuşoglu et al., 2007), enzyme activities (Cakmak, 2005), and the assimilate transport in the photosynthetic process. However, Ca^2+^ has a remarkable potential to maintain membrane stability (Hirschi, 2004; Tuna et al., 2007) and initiate signal transduction pathway to improve salt-adaptation (Zhu, 2003; Mahajan et al., 2008). To cope with salt toxicity, numerous gene activities are induced (Kreslavski et al., 2009; Shapiguzov et al., 2015), for instance the H^+^-ATPase which generates the proton-motive force for driving Na^+^/H^+^ exchanger (*NHX1*) and the *CAMK2*, a downstream factor sensing calcium signal (Zhu, 2003).

Tall fescue, a cool-season perennial species of turfgrass and forage, is widely used in the temperate zones. However, regional salinization limits tall fescue extensions on account of its damage on turf persistence and forage yield (Cao et al., 2009). Previous studies reported that exogenous calcium ameliorated salinity stress in tall fescue (*Festuca arundinacea*; Zhu et al., 2007), sheep grass (*Aneurolepidium chinense*), and reed canarygrass (*Phalaris arundinacea* L.; Maeda et al., 2003). However, the mechanism of Ca^2+^ alleviating the damage of salinity to PSII photochemistry has not yet been clearly studied. The aim of this study was to uncover the difference in mechanism by which exogenous calcium application lead to the rearrangements of PSII photochemistry and ion accumulation in tall fescue under salt stress.

## Materials and methods

### Plant materials and growth conditions

Single clonal plants of tall fescue genotype “TF133” were employed. Tall fescue tillers were initially transplanted from field plots to plastic containers (13 cm diameter, 11 cm deep) filled with a commercially available plant medium (general type, Zhenjiang Peilei Organic Fertilizer Co., Ltd., Jiangsu, China) and cleaned sand. 300 ± 10 g medium and 500 ± 10 g sand were used. The plants were maintained in a controlled greenhouse with natural sunlight (240 μmol m^−2^s^−1^), day/night temperature of 22/18°C, and average relative humidity of 70%. The plants were fertilized twice weekly with half-strength Hoagland's solution (1/2 HS) and mowed weekly to a height of 7 cm. The half-strength Hoagland's solution components were given per liter as follow, NH_4_H_2_PO_4_ (0.5 mM), KNO_3_ (2.5 mM), Ca(NO_3_)_2_.4H_2_O (2.5 mM), MgSO_4_.7H_2_O (1 mM), H_3_BO_3_ (1.43 mg), ZnSO_4_.7H_2_O (0.11 mg), CuSO_4_·5H_2_O (0.04 mg), MnCl_2_.4H_2_O (0.91 mg), H_2_MoO_4_ (0.05 mg), Fe-EDTA (0.04 mM) commercially available.

After 3 month establishment of canopy and root, the plants were thoroughly rinsed in distilled water and transferred into 300 mL Erlenmeyer flasks which were filled with ~290 mL 1/2 HS. The flasks were covered by aluminum foil and the bottlenecks were stuffed with appropriate amount of absorbent paper twined using food preservative film to prevent any algal growth. To protect plants from the hypoxia, each flask introduced 0.1 mM magnesium oxide for supplying additional oxygen and 1/2 HS was replaced every second day. The plants were kept in growth room with daily temperature of 22/18°C (day/night), 70% relative humidity, photosynthetically active radiation (PAR) at 300 μmol m^−2^s^−1^ and 14/10 h photoperiod, plants in the hydroponic systems were permitted to acclimate 2 weeks before the treatments were initiated.

### Treatments and experimental design

After 2-weeks of pre-adaptation, plant-flask systems were respectively weighed at 0 and 48 h to determine transpiration rate (Tr) according to the method described by Hu et al. (2011). Treatments were classified as the control (CK), salinity (S), salinity +calcium (SC), and salinity+ ethylene glycol tetraacetic acid (SE). Each treatment was replicated three times. Plant-flask systems with similar Tr were arranged in the same replicate, In the control, tall fescue plants were allowed to grow in 1/2 HS throughout the entire experimental period. Plants in S group were treated with 300 mM sodium chloride (Hu et al., 2013, 2016). Furthermore, plants in group SC and SE were subjected to 7 mM of calcium nitrate and 1 mM of ethylene glycol tetraacetic acid (EGTA). Calcium nitrate was used as exogenous calcium donor to increase calcium content in the nutrient solution according to the previous reports (Murillo-Amador et al., 2006; Tian et al., 2015), whose effect was reversed by calcium chelating agent (EGTA). The leaf samples for physiological assay were harvested at 0 and 5 d after treatment began. Gene transcription levels in the shoots were quantified at 1 and 5 d. The root samples for the above determination were collected at 5 d alone to avoid mechanical injury during this time.

### Chlorophyll *a* fluorescence transient and the JIP-test

Chlorophyll *a* fluorescence transient was conducted by a pulse-amplitude modulation fluorometer (PAM2500, Heinz Walz GmbH). After dark-adaptation for 30 min, the shoots (fully expanded 3rd) were exposed to a red light of 3,000 μmol photons m^−2^ s^−1^ (sufficient excitation intensity to assure the closure of all PSII reaction centers to obtain true fluorescence intensity of F_m_) supplied by an array of light-emitting diodes. Fluorescence emission between 10 μs and 300 ms was digitized to depict chlorophyll fluorescence kinetics curve. Each treatment was replicated three times.

The JIP test was capable of translating the primary data into biophysical parameters based on the theory of energy fluxes in biofilm (Force et al., 2003). The extracted parameters (F_m_, F20 μs, F300 μs, F_J_, F_I_ etc.) then led to the calculation and deduction of a variety of new parameters according to pervious authors (Yusuf et al., 2010). Details of the presented parameters are listed in Table 1.

**Table 1:** Photosynthetic parameters deduced by the JIP-test analysis of fluorescence transients.

	**0 DAY**				**5 DAY**				**Definitions**
	**SE**	**S**	**SC**	**CK**	**SE**	**S**	**SC**	**CK**	
**DATA EXTRACTED FROM THE RECORDED OJIP FLUORESCENCE TRANSIENT CURVES**									
F_0_ = F_20μ*s*_	0.46a	0.47a	0.48a	0.49a	0.45a	0.46a	0.47a	0.49a	Fluorescence at time t after onset of actinic illumination
F_K_	1.05a	1.08a	1.07a	0.98b	1.12ab	1.16a	1.13ab	1.05b	Fluorescence value at 300 μs
F_J_	1.25a	1.25a	1.27a	1.19a	1.27a	1.30a	1.31a	1.24a	Fluorescence value at the J-step of OJIP
F_I_	1.70a	1.60b	1.73a	1.69a	1.65b	1.70ab	1.73a	1.70ab	Fluorescence value at the I-step of OJIP
F_P_ = F_M_	1.89a	1.86a	1.95a	1.89a	1.79b	1.87ab	1.92a	1.90a	Fluorescence value at the peak of OJIP test
M_0_	1.61a	1.77a	1.61a	1.44b	2.01a	1.99a	1.82b	1.60c	Approximate value of the initial slope of fluorescence transient curves
V_J_	0.55ab	0.56a	0.54ab	0.51b	0.61a	0.60ab	0.58b	0.53c	Relative variable fluorescence at J-step
Area	10.45a	15.41a	12.86a	10.45a	7.38b	11.82ab	14.43a	11.99ab	The area above the chlorophyll fluorescence curve between Fo and Fm
N	21.96a	34.66a	26.11a	20.91a	17.99b	27.82ab	31.52a	25.38ab	Number of Q_A_ redox turnovers until Fm is reached
**SPECIFIC ENERGY FLUXES (PER ACTIVE PSII REACTION CENTER)**									
ABS/RC	0.65ab	0.75a	0.66ab	0.55b	0.93a	0.90a	0.79b	0.63c	Absorbed photon flux per RC
TR_0_/RC	2.96b	3.16a	2.98b	2.84b	3.27ab	3.34a	3.16bc	3.00c	Trapped excitation flux (leading to Q_A_ reduction) per RC
ET_0_/RC	1.35a	1.39a	1.37a	1.39a	1.27b	1.34ab	1.33ab	1.40a	Electron transport flux (further than ${\text{Q}}_{\text{A}}^{-}$) per RC
RE_0_/RC	0.08a	0.12a	0.09a	0.09a	0.05c	0.07bc	0.07ab	0.10a	Electron flux reducing end electron acceptors at the PSI acceptor side, per RC
**QUANTUM YIELDS AND EFFICIENCIES/PROBABILITIES**									
φP_0_ = TR_0_/ABS	0.74a	0.75a	0.75a	0.75a	0.75a	0.75a	0.75a	0.74a	Maximum quantum yield for primary photochemistry
ψE_0_ = ET_0_/TR_0_	0.45ab	0.44b	0.46ab	0.49a	0.39c	0.40bc	0.42b	0.47a	Efficiency/probability with which a PSII trapped electron is transferred from Q_A_ to Q_B_
φE_0_ = ET_0_/ABS	0.34b	0.33b	0.35ab	0.37a	0.29c	0.30bc	0.32b	0.35a	Quantum yield of the electron transport flux from Q_A_ to Q_B_
σR_0_ = RE_0_/ET_0_	0.06b	0.08a	0.07ab	0.07ab	0.04c	0.05bc	0.05ab	0.07a	Efficiency/probability with which an electron from Q_B_ is transferred until PSI acceptors
φR_0_ = RE_0_/ABS	0.10b	0.14a	0.11b	0.10b	0.07b	0.09ab	0.10ab	0.11a	Quantum yield for reduction of end Electron acceptors at the PSI acceptor side
γRC	0.20ab	0.19b	0.20ab	0.21a	0.18b	0.19ab	0.19ab	0.20a	Probability that a PSII Chl molecule functions as RC
RC/ABS	1.53b	1.35b	1.54b	1.82a	1.08c	1.11c	1.26b	1.58a	Number of Q_A_ reducing RCs per PSII antenna Chl
**PERFORMANCE INDEXES (PI, COMBINATION OF PARAMETERS)**									
PI_ABS_	0.49b	0.38b	0.45b	0.61a	0.35b	0.38b	0.43ab	0.51a	PI (potential) for energy conservation from exciton to the reduction of intersystem electron
PI_total_	0.03a	0.04a	0.04a	0.04a	0.01b	0.02b	0.03b	0.04a	PI (potential) for energy conservation from exciton to the reduction of PSI end acceptors

*Each parameter is carefully calculated according to previous method (Yusuf et al., 2010). Subscript “0” denotes that the parameter refers to the onset of illumination. Values are given as the average of 3 replicates, and different letters denote statistic significant difference at P < 0.05 among the treatments by Tukey's multiple range tests. 0 and 5 day, respectively executed variance analysis. The underline values are all carefully selected to avoid Blindness on browsing miscellaneous data*.

### Chlorophyll content and electrolyte leakage

Leaf chlorophyll content was quantified using SPAD 502 Plus Chlorophyll Meter (SPAD-502Plus, Spectrum Technologies, Inc., USA; Coste et al., 2010). In detail, three leaves (*in vivo*) were selected randomly from each treatment to form core collection. To determine electrolyte leakage (EL), 0.15 g of fully expanded fresh leaves were washed with deionized water and cut to about 0.5 cm long segments. Then, the leaves were transferred into a 50 mL test tube filled with 15 mL of deionized water, shaken for 24 h at 25°C until the initial conductivity (C_i_) was measured using a conductivity meter (JENCO-3173, Jenco Instruments, Inc., San Diego, CA, USA). Subsequently, the samples were autoclaved at 121°C for 30 min to release all electrolytes completely (Hu et al., 2012). The final conductivity (C_max_) was determined after the incubation solution cooled down to room temperature. Relative EL was calculated as (C_i_/C_max_) × 100%.

### Determination of C, N percentage composition, and ion content

To determine the C, N, Ca^2+^, and K^+^ concentration, the fresh shoots (0.3 g) and roots (0.3 g) samples were cautiously washed with deionized water, put into oven at 105°C for 30 min and dried to constant at 80°C. The dry plant samples were then ground in 1.5 mL Eppendorf tubes with high-throughput plant tissue ball mill instrument (Scientz-192, Xinzhi Biotechnology Co., Ltd., Ningbo, China).

The total C and N content in plant was measured by an isotope ratio mass spectrometry (IRMS) (Delta v advantage, Thermo Fisher Scientific, Germany) using carbamide as standard substance (Tomaszek, 2005). Weighed and sealed into tin capsules, ~0.2 and 0.4 mg samples of shoots and roots were loaded into an automatic sampler for IRMS analysis. Values were expressed as percentage composition.

The remaining samples were subjected to wet digestion with a mixture of HNO_3_, HCl, HF at 5:2:2 (V/V). The K^+^ and Ca^2+^ content of the mineral extract were determined by atomic absorption spectroscopy (PerkinElmer, Optima 8000DV, America) with standard sample (National Institute of Metrology, Beijing, China). The concentration of Ca^2+^, K^+^ was defined as the Ca^2+^, K^+^ content (mg) per unit tissues (g) (Li et al., 2017).

### Quantitative RT-PCR analysis

Total RNA was isolated from about 0.1 g leaves and roots using Trizol reagent (Invitrogen, America) and treated with RNase-free DNaseI to eliminate the contaminating genomic DNA. The concentration and quality of RNA preparations were both examined by spectrophotometry (UV-2600, UNICO Instruments Co., Ltd., Shanghai, China) and gel electrophoresis in 1.5% agarose gels.

For real time (RT)-PCR analyses, 2 μg purified RNA was reversely transcribed using cDNA synthesis kit (Fermentas, Canada) with an oligo(dT)_12−18_ primer. The resultant cDNA was diluted tenfold and kept at −20°C. The primers of two interested genes (Table 2) were synthesized based on previous reports (Hu et al., 2014). Then the qRT-PCR was conducted with ABI stepone plus real-time PCR system (Applied Biosystems, FosterCity, CA) and SYBR Green real-time PCR master mix (Toyobo, Japan) in 20 μL reactions to quantify the expression level of the target genes. The thermal cycles were programmed as follows: initial 3 min at 95°C, 38 cycles of 10 s at 94°C, 20 s at 50–55°C, and 20 s at 72°C, final 5 min at 72°C. At the end of each PCR reaction, melting curve examination of amplified products was performed to verify the presence of a single PCR product. *Tub* gene was used as the reference and comparative Ct method was applied in this analysis (Hu et al., 2013).

**Table 2:** Primer sequences for RT-PCR amplification analysis in tall fescue.

**Gene**	**Primers Sequences (5′-3′)**	**Accession**
*ATP6E*	F CTGTGGAGGCATTGAGGT	GBYN01013990
	R CGCAGACACGAGGAATAAC	
*CAMK2*	F CCAGAGGTTCTAAGGAAGGA	GBYN01000460
	R CGTGGAGCGATGTGAGAT	

*Gene sequences derived from the transcriptome data of tall fescue (Hu et al., 2014) and their accession was given as above*.

### Statistical analysis

All of above tests had three independent replicates. The data was subjected to analysis of variance (ANOVA) with the Tukey's multiple range tests means at a significant level of *P* < 0.05 using the statistical package SPSS (Version 20.0; IBM Corp., Armonk, NY), Origin Pro 9.0 and Excel 2010 for Windows. Results were expressed as mean ± *SD*, and letters in tables and histograms show significant differences between treatments in same category (same tissue at the same time).

## Results

### OJIP fluorescence transient and parameter analysis

**Figure 1:**
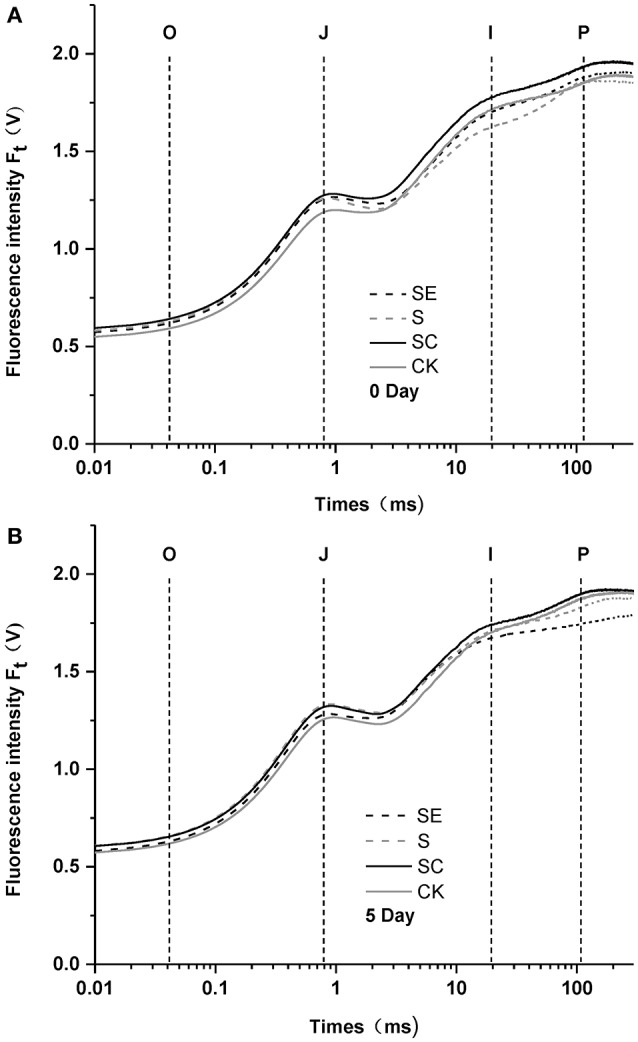
Polyphasic rise of chlorophyll fluorescence in tall fescue leaves before **(A)** and after **(B)** subjected to salt stress with different calcium regimes. S (Salt stress) and SC (Salt stess combined with exogenous calcium application) shows no difference when compared to CK 5 days later, while SE (Salt stess combined with calcium chelator, EGTA) revealed a weakened P-step **(B)**.

To investigate the role of calcium in tall fescue against salt stress, the responses of chlorophyll *a* fluorescence transient of tall fescue subjected to different calcium regimes were plotted in Figure 1. After 5-day treatment, tall fescue in the SC regime exhibited the best performance against salt stress while the SE treated tall fescue showed a great P-step depression and had the lowest Fm values. This gap between the two had been visibly enlarged after the treatment. Besides, the other three groups had not remarkable differences between 0 and 5 d. The ordinal phase transitions of chlorophyll a fluorescence transient mean the flow of electron or the reduction of ordered receptors on the thylakoid membranes of chloroplasts (Najafpour and Allakhverdiev, 2015). The step O to J was regarded as the reduction of Q_A_ by PSII, then rise to I phase, owing to the brimming plastoquinone pool, and the step I to P was account for the block of electron transfer to the acceptor side of PSI. The results suggested that calcium deficiency led to the photosynthetic electron transport traffic jam, especially beyond ${\text{Q}}_{\text{A}}^{-}$.

Further data analyses were prudently conducted to look into the authenticity. Basic fluorescence parameters and other structural and functional parameters were used to quantify the photosynthetic behavior of the samples (Table 1). The definitions of these parameters had been detailed in the Table 1.

**Figure 2:**
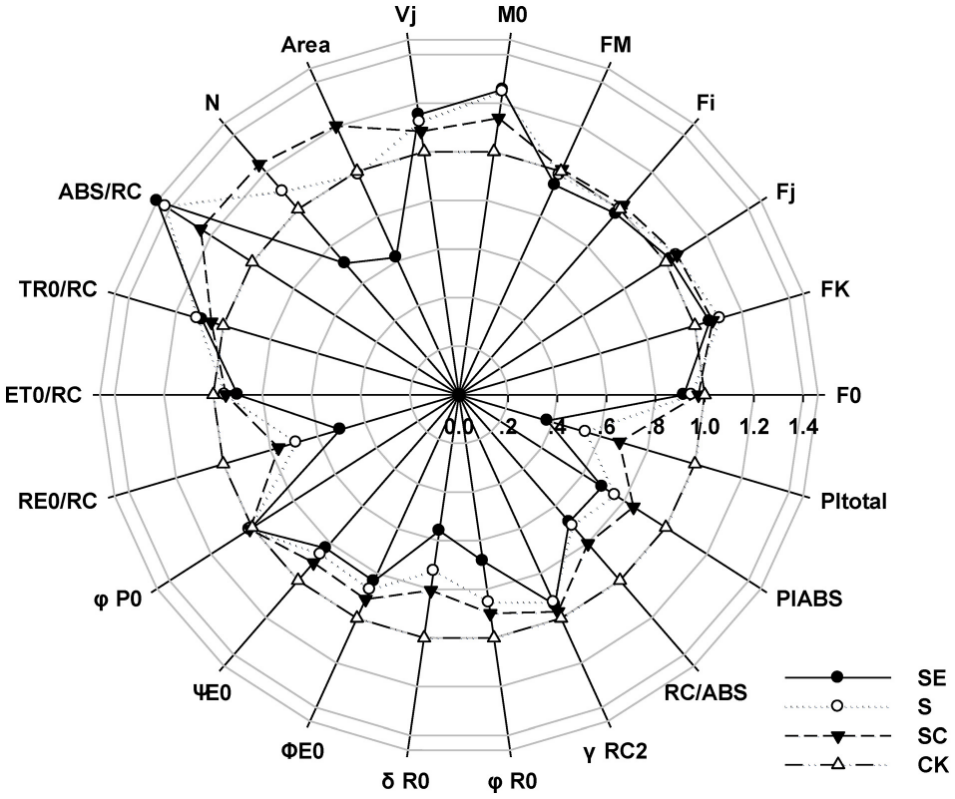
A “radar plot” of picked parameters characterizing different behavior of Photosystem II of tall fescue leaves exposed to diverse saline environment. All values are shown as percent of control (control plants = 1).

Frist, we considered the indexes representing the overall activity of PSII (PI_total_ and PI_ABS_), both of them were significantly decreased by salt stress and more aggravated by SE treatment, however, SC treatment improved it (Figure 2). It suggested that calcium played a positive role on the whole photosynthetic performance of tall fescue under salt stress. Besides, exogenous calcium application induced a greater level of Area, which represented the size of plastoquinone pool, and promoted energy fluxes (ET_0_/RC, RE_0_/RC; Table 1, Figure 2), and electron transport efficiency or quantum yield beyond ${\text{Q}}_{\text{A}}^{-}$ (ψE_0_, φE_0_, σR_0_, φR_0_; Table 1, Figure 2). In contrast, the absence of exogenous calcium decreased above parameters when subjected to salt stress. It demonstrated that exogenous calcium facilitated the photosynthetic electron transport beyond ${\text{Q}}_{\text{A}}^{-}$.

It is noteworthy that the ABS/RC level of S and SE treatment was increased much more significantly than SC treatment (Table 1, Figure 2). In the meantime, the maximum quantum yield for primary photochemistry (φP_0_) presented no changes. In other words, the amount of quanta absorbed per unit time was almost constant. Thus, we proposed that PSII reaction centers were inactivated more drastically in the absence of calcium and calcium adding helped the tall fescue to defend against that inactivation.

### Chlorophyll content and electrolyte leakage

**Figure 3:**
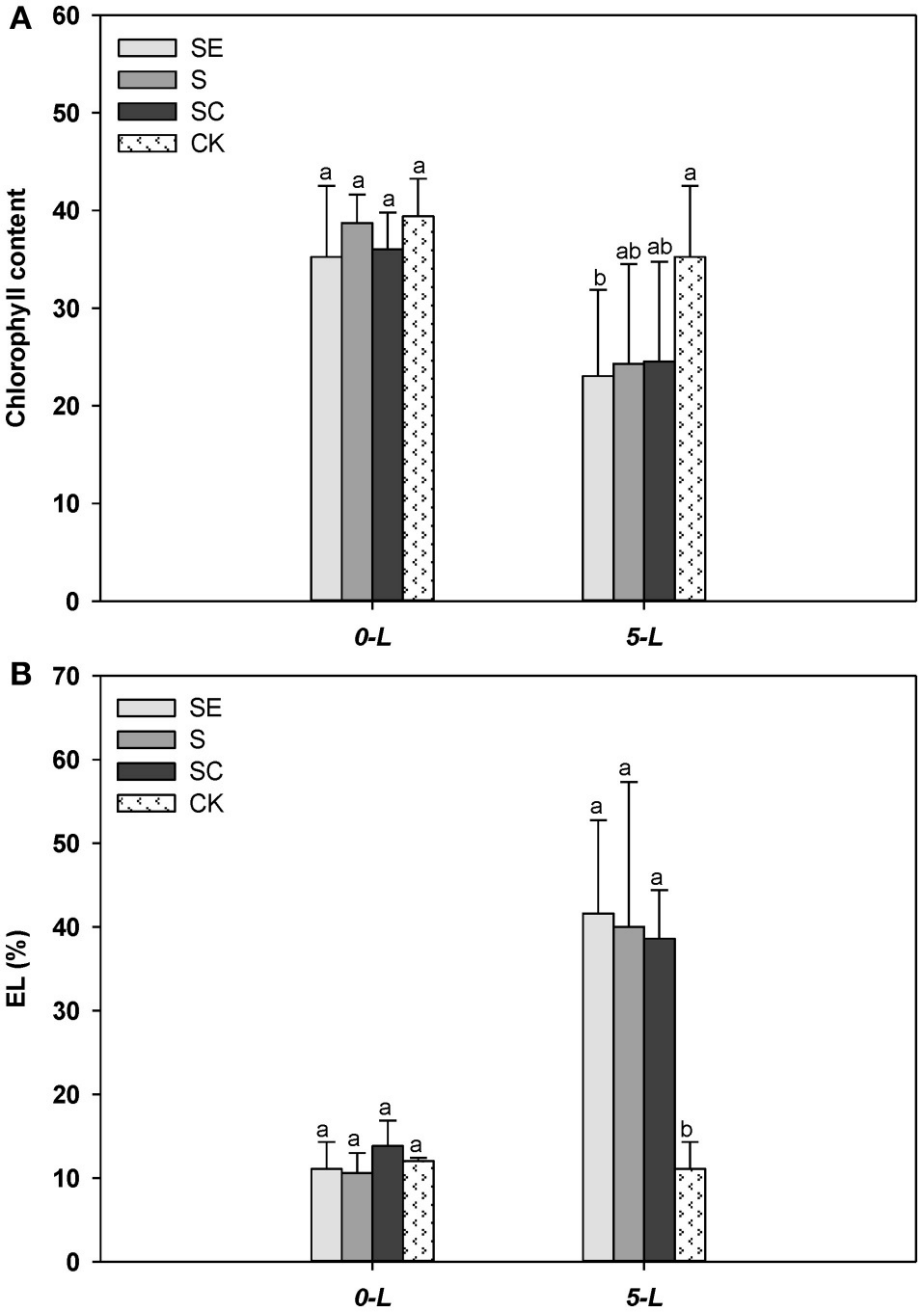
Calcium addition gently alleviated the reduction of chlorophyll content **(A)** and electrolyte leakage upsurge **(B)**. Three independent experiments were performed in chlorophyll content determination specially. Different letters above the same columns indicate statistic significant difference at *P* < 0.05 by Tukey's multiple range tests. Comparisons were carried out among the same tissue at same time, respectively.

Tall fescue generally exhibited less chlorophyll (Figure 3A) and a greater level of the EL (Figure 3B) compared with the control when subjected to salinity stress at 5 d. However, no difference in chlorophyll content and EL was observed among three salinity regimes (Figures 3A,B). The sharp upsurge in electrolyte leakage and detectable drop in chlorophyll content showed salt injury to the samples. However, the effect of calcium was mild on resisting this damage among treatment groups which may be due to the short duration of stress or the diversity of genotypes (Yasar et al., 2008; Sevengor et al., 2011).

### Total C, N, Ca^2+^, and K^+^ accumulation in shoots and roots

**Figure 4:**
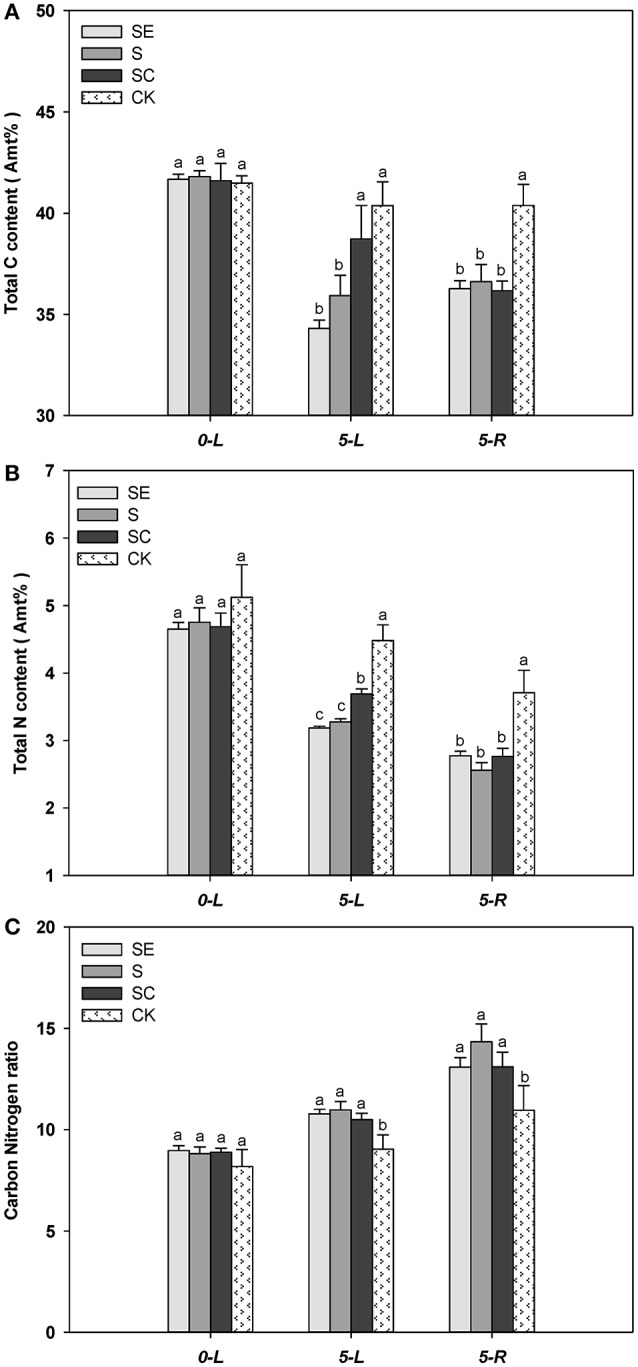
Exogenous calcium facilitated the carbon **(A)** and nitrogen **(B)** assimilation of tall fescue in salt environment. However, it showed no significant effect on Carbon Nitrogen ratio **(C)**. Columns marked with different letters indicate statistic significant difference at *P* < 0.05 by Tukey's multiple range tests. “0-L” and “5-L” on the horizontal axis means experiments are carried out in shoots at 0 and 5 days after treatments (DAT), similarly, “5-R” represents roots at 5 DAT. Comparisons were carried out among the same tissue at same time, respectively.

When experiment began, tall fescue leaf had similar total C percentage for all four regimes. The total C percentage in tall fescue leaf and root was lower when subjected to salt stress for 5 days (Figure 4A). SC treated tall fescue leaf had a greater level of total C percentage compared to SE and S treated shoots at 5 d (Figure 4A). There was no difference in total percentage between SE and S regimes (Figure 4A). The total carbon content in the shoots was lower by 17.68, 14.08, and 6.90% in SE, S, and SC groups at 5 days, respectively. However, total C percentage in root was similar among three regimes at 5 d (Figure 4A).

Similarly, nitrogen content in leaf and root also declined at 5 d vs. at 0 d, calcium treated shoots had a higher level of N content compared to S and SE treated shoots, and no difference in N content was observed among three salinity treatments in root (Figure 4B). However, greater reduction in N content for all three salinity regimes emerged when compared to the change of C content. In addition, Salinity stressed tall fescue leaf and root had a greater level of C/N ratio than the untreated tall fescue. However, C/N ratio was similar among three salinity regimes (Figure 4C).

Generally, the carbon and nitrogen assimilation was highly related to the whole photosynthesis process, because several photosynthetic intermediate products satisfied the needs of the anabolic reaction, such as reducing power and carbon skeleton. As we discussed above, calcium adding under salt stress had positive effects on the photosystem II photochemistry. Thus, the increased total C, N percentage composition in SC group can be due to the impact of exogenous calcium. It also implied why calcium had no effect on roots, a non-photosynthetic tissue.

**Figure 5:**
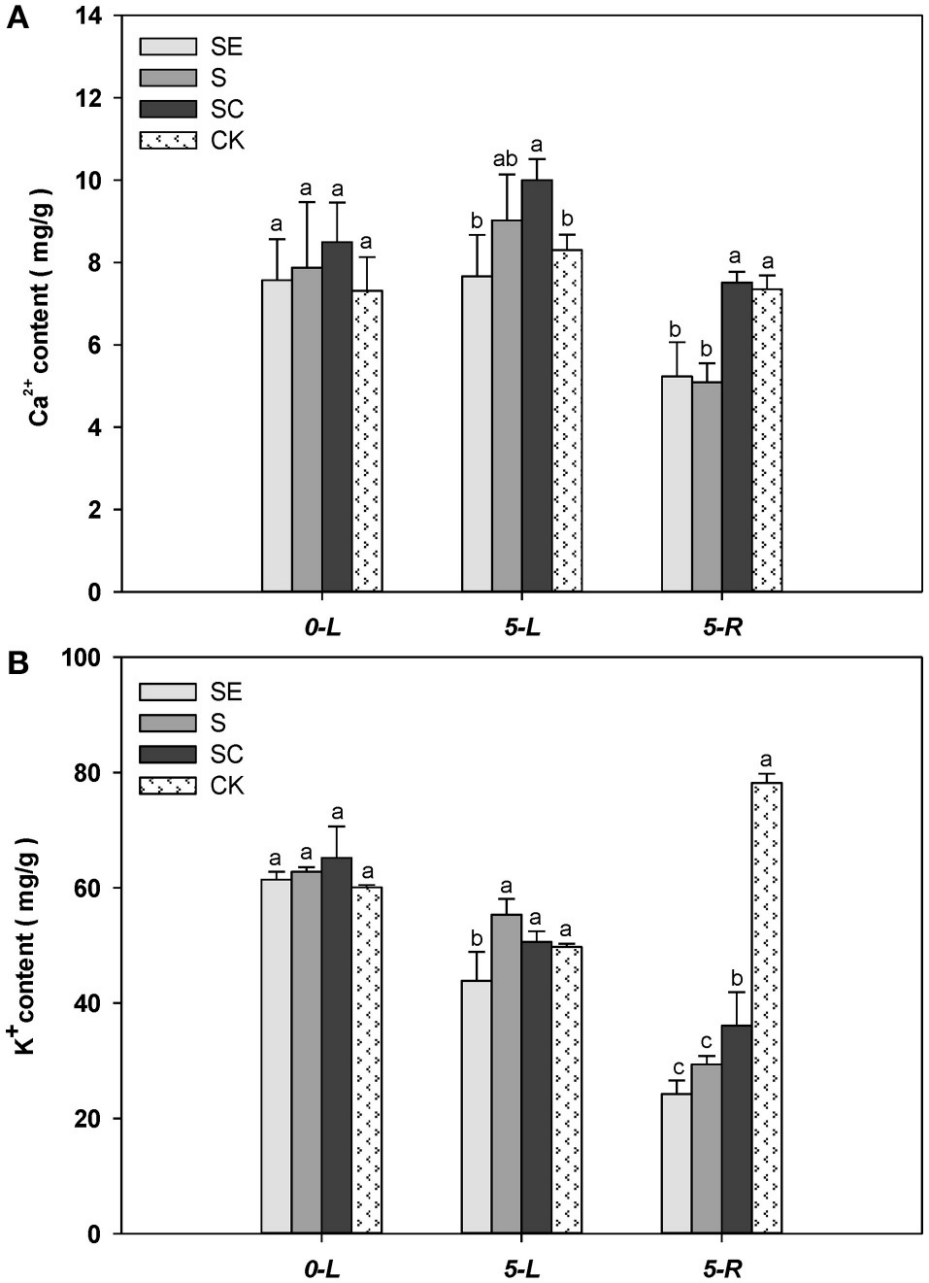
The quantification of calcium, potassium content in shoots and roots Calcium concentration gradient in shoots was actually confirmed **(A)**. In addition, the potassium absorption was promoted by calcium application **(B)**, which was obviously reflected in roots. Columns marked with different letters indicate statistic significant difference at *P* < 0.05 (Tukey's multiple range test). Comparisons were carried out among the same tissue at same time, respectively.

The Ca^2+^ content in shoots was greater at 5 vs. 0 d (Figure 5A). When subjected to salinity for 5 days, SC treated tall fescue shoots accumulated more Ca^2+^ than ones in the SE and the control regimes. Although, difference in Ca^2+^ content in shoots was not significant between the SE and the S regimes, the Ca^2+^ content in the root was greater in the SC and the CK regimes vs. the SE and the S regimes. The root generally had less Ca^2+^ content than the shoot tissues. No difference in root Ca^2+^ content was observed between the SE and the S regimes as well as between the SC and the control regimes. The existence of calcium concentration gradient in shoots and roots demonstrate the effectiveness of the experimental design while the gradient difference had not got predicted objectives. It may be mainly due to calcium itself which is obtained and subsequently transported to shoots in the form of ion and susceptible to high salt. Whether the effect would be increased under prolonged stress is still not clear and warrants further research.

When subjected to salinity for 5 days, tall fescue shoot tissues generally accumulated less K^+^ regardless of the treatments (Figure 5B). The SE treated tall fescue shoots accumulated the least K^+^ content compared to the other three regimes. In roots, K^+^ level were lower than the shoots subjected to the three salinity treatments. The calcium application maintained a greater level of K^+^ content than the other two treatments under salinity conditions. It was suggested that exogenous calcium enhanced the uptake of K^+^ under salt stress. However, a moderate amount of K^+^ in cytoplasm was vital for tall fescue to survive in saltine condition.

### Gene expression induced by NaCl in shoots and roots

**Figure 6:**
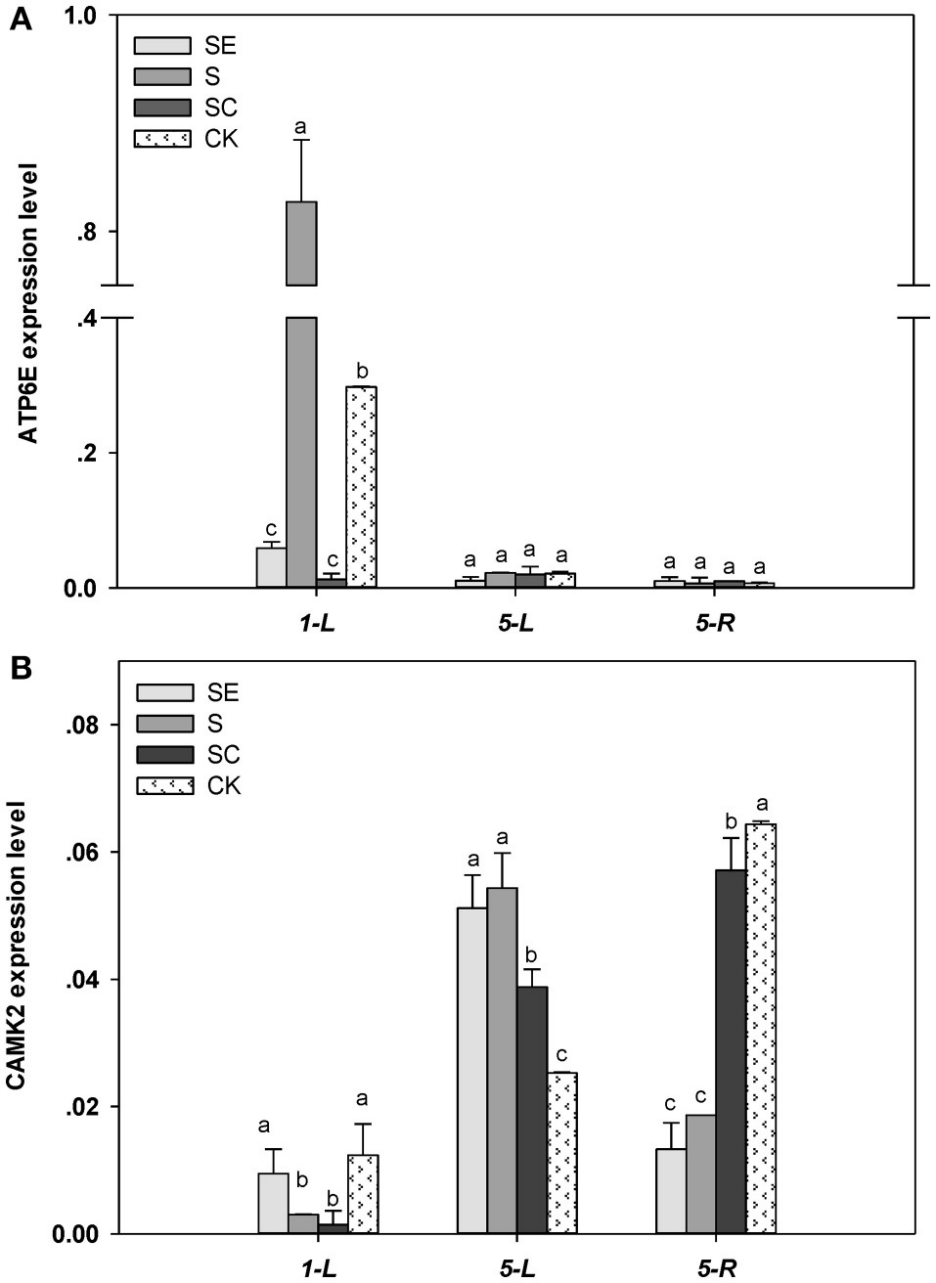
*ATP6E* **(A)** and *CAMK2* **(B)** transcriptional level in tall fescue exposed to salinity. Different letters above the same columns indicate statistic significant difference at *P* < 0.05 (Tukey's multiple range test). Calcium led to a 95.8% reduction in the ATP6E expression than the control under salinity stress conditions. Comparisons were carried out among the same tissue at same time, respectively.

The expression of *ATP6E* in the shoots was higher by 178% for the S regime vs. the control regimes at 1 day (Figure 6A). However, EGTA and calcium application led to an 80.3, 95.8% reduction in the *ATP6E* expression than the control under salinity stress conditions, respectively. Both shoots and roots exhibited a similar expression of *ATP6E* in response to different calcium regime at 5 days.

Under salt stress conditions, the transcription level of *CAMK2* in the shoots declined at 24 h and then, increased at 5 d (Figure 6B). *CAMK2* expression was similar between the SE and the S regimes, but greater compared to the SC regime and the control. The root subjected to salinity for 5 d had a lower expression of *CAMK2* in the SE and S regimes vs. the SC regimes and the control. Non salinity stressed root exhibited the greatest expression of *CAMK2*.

In shoots, both *ATP6E* and *CAMK2* transcription level were increased by high salt while relieved by exogenous calcium, but *ATP6E* seem to be activated earlier than *CAMK2*. In roots, *ATP6E* level had no difference among treatments, but *CAMK2* level was declined by salt stress and remedied by calcium addition.

### Relationships between photosynthetic parameter and physiological index

Any two of ψE_0_, δRo, V_J_, ABS/RC, and M_0_ presented significant correlation (*P* < 0.01) and ψE_0_ and δRo negatively correlated to the other three parameters (Table 2). C and N percentage composition was significantly positively related to K^+^ and Ca^2+^ content in roots. However, the relationship in the shoots was not remarkably related (*P* < 0.05). Besides, profound correlations were found between C and N percentage in leaf or K^+^ and Ca^2+^ concentration in root. Physiological indexes and photosynthetic parameters also revealed significant correlations. The C and N percentage were positively correlated with ψE_0_, δRo, and negatively related to V_J_, ABS/RC, and M_0_, while both reached the significant level (*P* < 0.01). Similar relationships were uncovered between K^+^ and Ca^2+^ content in roots and these photochemical parameters.

## Discussion

### Exogenous calcium facilitated the photosynthetic electron transport beyond ${\text{Q}}_{\text{A}}^{-}$

Salinity has initial impact on complexes of photosystem II (Aro et al., 1993). Shown in the polyphasic rise of chlorophyll fluorescence, a weakened P-step was observed under high salinity combined with calcium deficit (Figure 1B). However, no significant change emerged when samples were treated with up to 300 mM/L of NaCl alone in this study. Govindje (1995) suggested that the O-J-I-P transients represented the sequential reduction of the electron acceptors of PSII. The J-P phase transition results from the electron transport from ${\text{Q}}_{\text{A}}^{-}$ to ${\text{Q}}_{\text{B}}^{2-}$ (Strasserf et al., 1995; Lazár, 1999). The data obtained in this study likely reflect that PSII in tall fescue rather tolerant to high salinity. Similar results were also detected in Rumex (Chen et al., 2004) and *Suaeda salsa* (Lu et al., 2003), and the target site of salinity stress was located at the acceptor side in the electron transfer chain of PSII when calcium was deficient.

To validate these assumptions, numerous parameters deduced from JIP-test were analyzed. The F_K_ and F_V_/F_0_ (no shown in table) values which were proportional to the efficiency of the water-splitting complex (Schreiber et al., 1994), showed no difference when treated with diverse calcium regime under salt stress. In addition, no obvious K-step was observed in fluorescence induction curve. These may be attributed to the weak impact of calcium at the donor side of PSII in salty environment. On the other hand, the area above the chlorophyll fluorescence curve between F_0_ and F_M_ significantly declined with the reduction in calcium concentration from SC to SE group. It was consistent with the performance of N (reduce times of Q_A_ while fluorescence reached its maximal value, which different from the abbreviation of nitrogen). The area and N decreases had been reported to be related to the block of electron transfer from reaction centers to quinine pool. Moreover, efficiency/probability with which a PSII trapped electron is transferred further than ${\text{Q}}_{\text{A}}^{-}$ (ψE_0_, σR_0_) and their quantum yield of the electron transport flux (φE_0_, φR_0_) were measured. The calcium shortage with salinity also dramatically pulled the level of these parameters down compared to those in calcium abundant regimes. Thus, we proposed that calcium played an essential role on protecting photochemistry activities from salt damage and functioned at the acceptor side in the electron transfer of PSII, especially beyond ${\text{Q}}_{\text{A}}^{-}$.

### Exogenous calcium protected the PSII reaction centers from inactivation

To investigate the functioning of reaction centers (RCs), relative variable fluorescence (V_J_), whose value was equal to the proportion of RCs could be closed at J-step (Force et al., 2003), was assessed. Results showed that salinity initiated the inactivation of RCs, and calcium deficiency further worsened this status. Nevertheless, maximum quantum yield for primary photochemistry (φP_0_) displayed no changes. It can be stipulated that the amount of quanta absorbed per unit time was not influenced in this case. According to the above analyses, it could have accounted for the increase in ABS/RC value and the accelerated reduction rate of Q_A_ (M_0_) (Strasser et al., 2004). It may be mainly because the speed of reduction was accelerated when constant quantum passed through the decreased reaction centers.

Based on the fact that the routine light quantum absorption was maintained under salinity while the electron transport beyond ${\text{Q}}_{\text{A}}^{-}$ was inhibited, we speculated that a reversible inactivation of RCs in PSII may have occurred (Allakhverdiev and Murata, 2004). Previous works had reported that the normal illumination became excess when plants were subjected to environmental stress, such as chilling injury (Allakhverdiev et al., 2003) and salt stress. However, fractional inactive RCs were able to effectively consume the excess excitation energy to protect themselves against the photo-inhibition (Allakhverdiev et al., 1997, 2007; Mohanty et al., 2007). The protective role of exogenous calcium under salt stress indicated the maintenance of unimpeded electron transfer and activated RCs to enhance the photosystem II photochemistry.

### Exogenous calcium promoted carbon and nitrogen assimilation in shoots, not roots

Almost all organic carbon in plants was generated by photosynthesis through CO_2_ fixation, which caused a demand for the assimilatory power coupled with photosynthetic electron transport (Lambers et al., 1998). This is probably because the traffic jam of electron transfer under salt stress resulted in the decline of total C percentage composition in the shoots in this study. Moreover, the total C content increase with increasing calcium level offered further evidence of the protective role of calcium on maintaining expedite electron transfer. Simultaneously with the chloroplast, nitrogen assimilation was closely associated with photosynthesis on basis of reducing power and carbon skeleton (Champigny, 1995). For instance, 6 electrons directly supplied by red-Fd met the demand of reducing ${\text{NO}}_{2}^{-}$ to ${\text{NH}}_{4}^{+}$ form (Kleinhofs and Warner, 1990), ATP coming from the “light reaction” provided energy for glutamine synthetase (GS) to catalyze the conversion from ${\text{NH}}_{4}^{+}$ to Gln (Vézina et al., 1987), and the formation of Glu from Gln not only required red-Fd (Huppe and Turpin, 1994) but also used oxoglutarate as carbon skeleton. The dramatically drop of nitrogen content in shoots could be strongly attributed to the inhibition of photochemistry activities. Nevertheless, no difference of carbon and nitrogen percentage composition in root tissue was observed with terraced calcium level under salinity. This likely implied that calcium played a more crucial role in chlorenchyma than non-photosynthetic tissue during photosynthesis when faced with salinity stress, and exogenous calcium facilitated carbon and nitrogen assimilation in tall fescue through enhancing photochemistry response to salt stress.

### Exogenous calcium enhanced the uptake of K^+^ under salt stress

In addition, the ion content quantifications verified the existence of calcium concentration gradient in shoots which was generated by the calcium chelating agent (EGTA) and exogenous calcium donor [CaNO_3_]. The K^+^ was also focused on in this test based on its diverse effects on regulating stoma conductance (Broadley et al., 2001; Çavuşoglu et al., 2007), inhibiting activity of NAD(P)H oxidases and sustaining photosynthetic electron transfer (Cakmak, 2005), and keeping ribulose bisphosphate carboxylase oxygenase (Rubisco) initial availability in photosynthetic process. Certain promoting effect of calcium on K^+^ uptake under salt stress was clearly noticed in roots where minerals absorption taking occurred directly. Associated with K^+^ content in roots, K^+^ level in shoots had similar status. It may be precisely because this indirect action of calcium enhanced carbon fixation and photosynthetic electron transport.

### Exogenous calcium alleviated the gene expression level changes

V-ATPase was functioned as a proton pump establishing proton-motive force to drive the transmembrane transport of ion and metabolites (Ratajczak, 2000) and widely distributed on the tonoplast and other endomembrane systems (Gaxiola et al., 2007). In this study, we considered the expression level of *ATP6E* as V-ATPase activity, also equal to the primary proton-motive force driving secondary transport, such as sequestrating redundant Na^+^ in vacuolar by NHX family (Silva and Gerós, 2009). However, Na^+^ compartmentation not only relieved ion toxicity in protoplast but also reconstructed the osmotic equilibrium to maintain water supply when plant suffered salt injury. It highlighted the significance of V-ATPase for plant survival in salt environment. So we proposed that tall fescue defend itself against ion disorder under salt stress and then increased the expression of *ATP6E*. Evidence suggested that calcium signal participate in the ABA- dependent pathway regulating the expression of *NHX1* in response to salt stress (Shi and Zhu, 2002). So we suggested exogenous calcium reinstated cellular ion homeostasis (Supplemental Figure 1) and decreased *ATP6E* expression. Besides, EGTA blocked the occurrence of Ca^2+^ signal and weakened the response to salinity initially. In addition, Calcium/Calmodulin-dependent protein kinase II (*CAMK2*), one type serine-threonine protein kinase belonged to the CDPK-SnRK superfamily (Hrabak et al., 2003), was assayed at transcriptional level. Previous researches suggested CDPK extensively participated in downstream cascades in salt stress-signaling (Boudsoc and Sheen, 2013; Schulz et al., 2013) and was increased at the gene expression level under salt conditions (Dubrovina et al., 2013). In this study, the level of *CAMK2* in shoots distinctly raised when subjected to salt at 5 days. But unlike V-ATPase subunit E, its response was more slowly may be result of the progressive salt damage. While the difference of *CAMK2* level between SC and SE group possibly due to the relief effect of exogenous calcium. For the root, high salt brought *CAMK2* level down and calcium effectively relieved it. Root is the tissue directly exposed to high salt and treatments, so we speculated *CAMK2* expression was suffered immediate damage of high salinity and calcium in solution acted directly on root cells.

### Fluorescence parameters were closely related to calcium content in roots

Close relationship between photosynthesis and calcium content were probed and displayed in Table 3. Exogenous calcium was expected to maintain smooth electron transfer and activate RC, although no significance of correlation coefficient was detected in shoots. This is probably because the calcium content in roots can more veritably reflect the whole calcium level in plants because the root was the organ functioning in mineral nutrition and water uptake directly or the ion upward transport and reaction happened with a lag. In addition, correlation analysis also revealed the interdependent relation between C and N assimilation, and promoting effect of calcium on the absorption of potassium in roots, which was highly related to photochemical parameters.

**Table 3:** Correlations between photochemical parameters and physiological indexes in tall fescue after 5-day treatments.

	**Area**	**N**	**Ψeo**	**δRo**	**V_J_**	**ABS/RC**	**M_0_**	**C-L**	**N-L**	**K-L**	**Ca-L**	**K-R**	**Ca-R**
Area	1.000												
N	0.944^**^	1.000											
Ψeo	0.503	0.399	1.000										
δRo	0.490	0.385	0.888^**^	1.000									
V_J_	−0.503	−0.399	−1.000^**^	−0.888^**^	1.000								
ABS/RC	−0.587^*^	−0.448	−0.979^**^	−0.874^**^	0.979^**^	1.000							
M_0_	−0.594^*^	−0.455	−0.965^**^	−0.811^**^	0.965^**^	0.979^**^	1.000						
C-L	0.671^*^	0.559	0.881^**^	0.755^**^	−0.881^**^	−0.909^**^	−0.923^**^	1.000					
N-L	0.524	0.427	0.888^**^	0.825^**^	−0.888^**^	−0.902^**^	−0.853^**^	0.895^**^	1.000				
K-L	0.175	0.350	0.042	0.175	−0.042	0.007	0.077	0.168	0.175	1.000			
Ca-L	0.783^**^	0.832^**^	0.413	0.399	−0.413	−0.434	−0.434	0.420	0.266	0.238	1.000		
K-R	0.524	0.378	0.874^**^	0.804^**^	−0.874^**^	−0.888^**^	−0.860^**^	0.916^**^	0.965^**^	0.140	0.224	1.000	
Ca-R	0.685^*^	0.531	0.685^*^	0.573	−0.685^*^	−0.748^**^	−0.762^**^	0.776^**^	0.685^*^	−0.231	0.559	0.706^*^	1.000

* *Indicates statistical difference significance at P < 0.05 among the treatments by Tukey's multiple range tests*.

** *Indicates statistical difference significance at P < 0.01 among the treatments by Tukey's multiple range tests*.

*L and R indicate leaves and roots of tall fescue of genotype TF133 respectively*.

*The underline values are all carefully selected to avoid blindness on browsing miscellaneous data*.

## Conclusion

The improved effect of exogenous calcium on photosystem II photochemistry activity against salt injury was emphasized in this work. It functions of smoothing the electron transfer of PSII (especially beyond ${\text{Q}}_{\text{A}}^{-}$) and maintaining reaction center activity. This protective role is further confirmed by a higher level of C and N fixation, K^+^ uptake induced by calcium.

## Author contributions

TH: ideated and designed the experiments; GW and AB: conducted the experiments and analyzed the data; GW: drafted the manuscript; TH, HL, EA, LZ, and JF: revised the manuscript. All authors have contributed to, approved the manuscript.

### Conflict of interest statement

The authors declare that the research was conducted in the absence of any commercial or financial relationships that could be construed as a potential conflict of interest.

## Supplementary material

Supplemental Figure 1 The quantification of sodium content in shoots and roots. The sodium absorption was reduced by calcium application while no obvious difference reflected in roots at 5 day. Columns marked with different letters indicate statistic significant difference at *P* < 0.05 (Tukey's multiple range test). Comparisons were carried out among the same tissue at same time, respectively.

## Abbreviations

*ATP6E*: V-type H^+^-transporting ATPase subunit E
C: carbon
*CAMK2*: calcium/calmodulin-dependent protein kinase II
EGTA: ethylene glycol tetraacetic acid
EL: electrolyte leakage
N: nitrogen
PSI: photosystem I
PSII: photosystem II
S: salt treatment
SC: salt treatment with exogenous calcium application
SE: salt treatment with ethylene glycol tetraacetic acid addition.
