# Supplementary figures and images for: Exogenous Calcium Enhances the Photosystem II Photochemistry Response in Salt Stressed Tall Fescue
Source: Front Plant Sci. 2017 Nov 30;8:2032. doi: 10.3389/fpls.2017.02032 (PMC5715236; doi:10.3389/fpls.2017.02032)

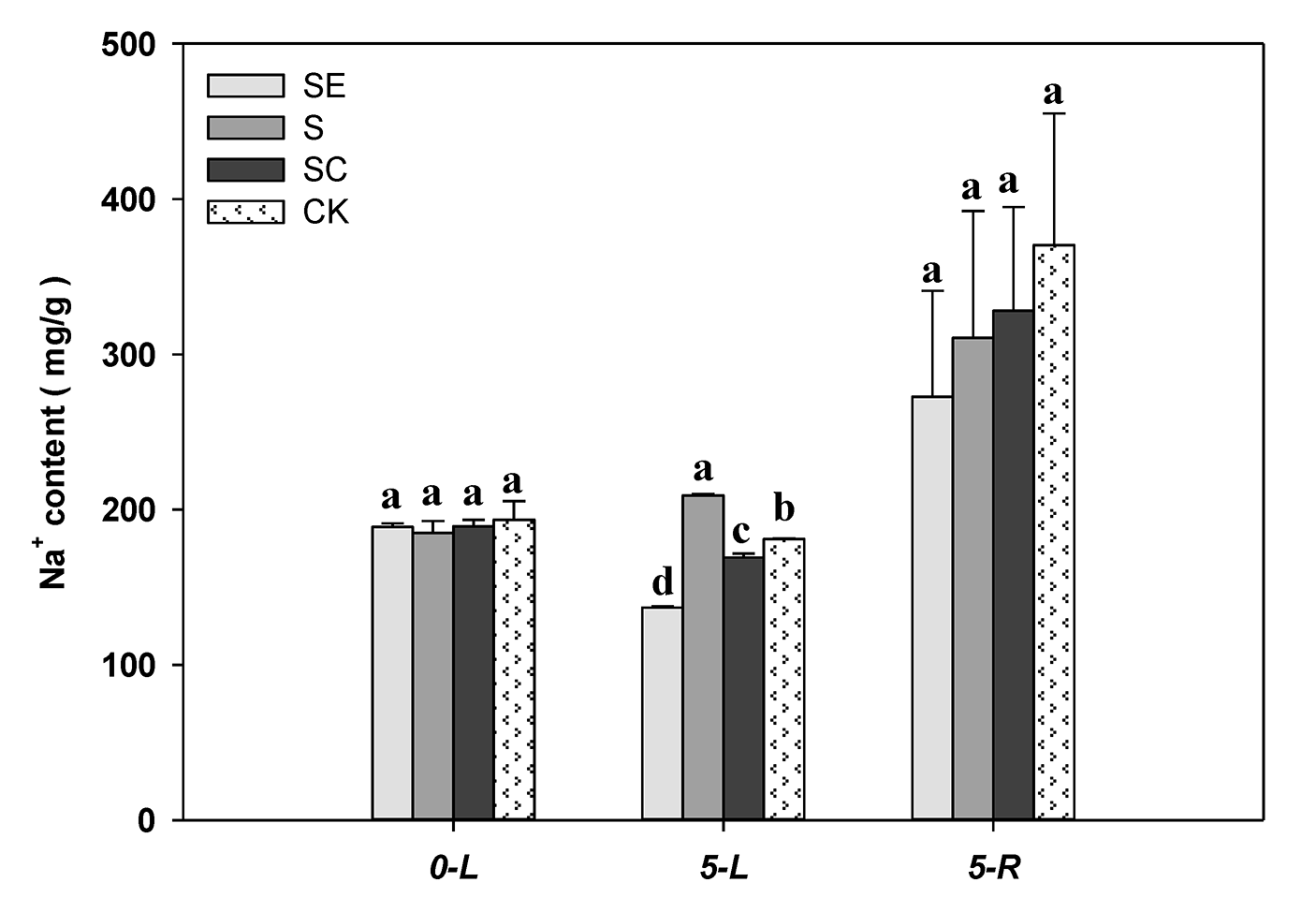

Supplement: Supplemental Figure 1 — The quantification of sodium content in shoots and roots. The sodium absorption was reduced by calcium application while no obvious difference reflected in roots at 5 day. Columns marked with different letters indicate statistic significant difference at P < 0.05 (Tukey's multiple range test). Comparisons were carried out among the same tissue at same time, respectively. [file Image1.TIFF]
